# Prevalence and factors associated with hypertension among older people living with HIV in South Africa

**DOI:** 10.1186/s12889-022-14091-y

**Published:** 2022-09-05

**Authors:** Joshua Okyere, Castro Ayebeng, Bernard Afriyie Owusu, Kwamena Sekyi Dickson

**Affiliations:** 1 Department of Population and Health, University of Cape Coast, Cape Coast, Ghana; 2 Department of Nursing, College of Health Sciences, Kwame Nkrumah University of Science and Technology, Kumasi, Ghana

**Keywords:** Hypertension, Risk factors, Older people, HIV, South Africa, Social Demography, Public Health

## Abstract

**Background:**

People living with HIV (PLHIV) are experiencing increased life expectancy mostly due to the success of anti-retroviral therapy. Consequently, they face the threat of chronic diseases attributed to ageing including hypertension. The risk of hypertension among PLHIV requires research attention particularly in South Africa where the prevalence of HIV is highest in Africa. We therefore examined the prevalence and factors associated with hypertension among older people living with HIV in South Africa.

**Methods:**

We analysed cross-sectional data on 514 older PLHIV. Data were extracted from the WHO SAGE Well-Being of Older People Study (WOPS) (2011–2013). The outcome variable was hypertension status. Data was analysed using STATA Version 14. Chi-square and binary logistic regression were performed. The results were presented in odds ratio with its corresponding confidence interval.

**Results:**

The prevalence of hypertension among PLHIV was 50.1%. Compared to PLHIV aged 50–59, those aged 60–69 [OR = 2.2; CI = 1.30,3.84], 70–79 years [OR = 2.8; CI = 1.37,5.82], and 80 + [OR = 4.9; CI = 1.68,14.05] had higher risk of hypertension. Females were more likely [OR = 5.5; CI = 2.67,11.12] than males to have hypertension. Persons ever diagnosed with stroke were more likely [OR = 3.3; CI = 1.04,10.65] to have hypertension when compared to their counterparts who have never been diagnosed with stroke. Compared to PLHIV who had no clinic visits, those who visited the clinic three to six times [OR = 5.3; CI = 1.35,21.01], or more than six times [OR = 5.5; CI = 1.41,21.41] were more likely to have hypertension.

**Conclusion:**

More than half of South African older PLHIV are hypertensive. The factors associated with hypertension among older PLHIV are age, sex, ever diagnosed with stroke and number of times visited the clinic. Integration of hypertension management and advocacy in HIV care is urgently needed in South Africa in order to accelerate reductions in the prevalence of hypertension among older PLHIV, as well as enhance South Africa’s capacity to attain the Sustainable Development Goal target 3.3.

## Background

Human immunodeficiency virus (HIV) continues to be a pandemic affecting millions of people worldwide. According to the Joint United Nations Programme on HIV/AIDS (UNAIDS), there are 38 million individuals living with HIV worldwide, with 1.5 million new infections in 2020 and nearly 6 million persons being unaware of their HIV status [1]. HIV is endemic in sub-Saharan Africa (SSA) where most people suffer the greatest burden of the disease [2, 3]. South Africa has the largest number of people living with HIV globally with an estimated 8 million people are living with HIV (PLHIV) in 2017 [4, 5]. To facilitate reduction in the incidence and prevalence of HIV, there have been global commitments such as the ended Millennium Development Goals (MDG), and the adopted Sustainable Development Goals (SDGs) target 3.3 which aims at ending HIV by 2030 [6]. These interventions have contributed to a significant decline in global HIV-related mortalities from a peak of 1.90 million in 2004 to 1.5 million in 2010 and 0.77 million in 2018 [7]. In South Africa, successful implementation of anti-retroviral therapy (ART) programme has also reduced HIV-related mortalities in the country [8]. Consequently, the effect of ART on viral load suppression has greatly improved due to ART has the life expectancy of PLHIV alongside a decline in opportunistic infections [8]. However, there has been an observed increase in hypertension among PLHIV. Improved understanding of factors associated hypertension among PLHIV is vital for designing tailored and targeted interventions [8–10].

Literature shows that the biology of HIV infection is such that there is pro-inflammatory effect on vascular endothelium which tends to significantly exacerbate PLHIV’s risk of hypertension [9, 11]. A related study [12] also postulates that ART, which is responsible for improving the health outcome and life expectancy of PLHIV increases the likelihood of having lower levels of high-density lipoprotein (HDL) cholesterol (i.e., good cholesterol), which tends to significantly increase the risk of hypertension among PLHIV. Thus, the occurrence of hypertension among PLHIV is undeniably intrinsic and varies across countries. In the United States for instance, the prevalence of hypertension among PLHIV is 67% [13]; in Uganda, the prevalence stands at 29% [14].

Beyond these biological risk factors, the question however remains whether socio-demographic, lifestyle and health-seeking factors have any association with respect to hypertension among PLHIV. Studies conducted in Nigeria [15], Malawi [16] and Ethiopia [17] indicate that place of residence, diabetes status, high body mass index, use of ART, alcohol consumption and ageing were significantly associated with higher risk of hypertension among PLHIV. People with hypertension are at high risk of other ill-health conditions including cardiovascular events, including arthrosclerosis, coronary disease, myocardial infarctions, and heart failure [18, 19]. Therefore, hypertension may adversely affect the quality of life of PLHIV. As such, evidence-based studies are needed to advance policy and planning intervention for the management of hypertension in HIV care. Yet, there is dearth of nationally representative studies that have examined the prevalence and factors associated with hypertension among older PLHIV in South Africa.

To the best of our knowledge, only one study [8] has examined the factors associated with hypertension among PLHIV in South Africa. However, Chiwandire et al.’s study [8] did not focus on the elderly or older people 50 years and older living with HIV in South Africa. Moreover, their study did not include residual confounders such as health-seeking behaviour. Hence, there are still gaps in what is known about the factors associated with hypertension among older PLHIV in South Africa. We, therefore, sought to examine the prevalence and factors associated with hypertension among older people living with HIV in South Africa.

## Methods

### Data source

In this study, older people are categorised as younger old (50–64), young old (65–74 years), old old (75–84 years), and the oldest old (85 years and above) [20]. Data utilised in this study were acquired from the WHO SAGE Well-Being of Older People Study (WOPS). These were population-based HIV surveys conducted in South Africa between 2010 (Wave 1) and 2013 (Wave 2) in collaboration with the Africa Centre Demographic Information System (ACDIS) [21]. The SAGE WOPS study gathers comparable longitudinal data on a variety of health, demographic, and social markers that are relevant to the health and functional status of older persons who are HIV-positive or have HIV/AIDS in their family [20]. In addition, the survey looked at the respondents’ nutritional status, and HIV treatment. Concerning the sampling method, the survey's sample was divided into five groups [20]. At the onset of Wave 1 of the project in 2010, the sample for Group 1 consisted of adults who had been receiving HIV therapy for at least a year. Aged individuals in Group 2 of Wave 1's 2010 cohort who were not receiving HIV therapy or who had only had it for three months or less. The third group of HIV-positive people in Wave 1 of 2010 were those who lived with adult (14–49-year-old) children. Group 4 was made up of elderly people who had experienced an HIV-related death of an adult household member in 2010. The aged who were not receiving HIV therapy or had only received it for three months or fewer in 2013 during Wave 2 were included in Group 5 [20]. The sampling methodology is described in detail elsewhere [22, 23].

### Measures

#### Outcome variable

The outcome variable is based on the question “Have you ever been diagnosed with hypertension”. The response option was "Yes" or "No", which has coded into a binary outcome with Yes = 1 and No = 0.

#### Independent variables

The following factors were identified and selected as explanatory variables based on literature review [15–17], and their availability in the dataset: age, sex, education, employment, body mass index (BMI), marital status, and household wealth index. Age was recoded as (0 = 50–59, 1 = 60–69, 2 = 70–79, 3 = 80 +), sex (coded 1 = male, 2 = female), level of education (recoded 0 = no formal education, 1 = basic, 2 = secondary +), employment (0 = not working, 1 = working), marital status (recoded 0 = married, 1 = divorced/separated, 2 = never married, 3 = widowed). Body mass index of respondents was calculated based on weight and height using standardised computation (0 = underweight, 1 = normal, 2 = overweight, 3 = obese), wealth index (0 = poorest, 1 = poorer, 2 = middle, 3 = richer, 4 = richest). Wealth index variable was computed from respondents’ source of water, toilet facility, cooking fuel, electricity, household assets, and having domestic animals using principal component analysis (PCA). PCA post estimation test was done with Kaiser–Meyer–Olkin of 0.7 indicating a good measure of sampling adequacy. Wealth index was then divided into five quintiles (1 = poorest, 2 = poorer, 3 = middle, 4 = richer, 5 = richest). The comorbidity variables were derived from the questions on whether a respondent has ever been diagnosed of the following health conditions: diabetes (0 = No, 1 = Yes), stroke (0 = No, 1 = Yes), arthritis (0 = No, 1 = Yes), asthma (0 = No, 1 = Yes), heart disease (0 = No, 1 = Yes), cancer (0 = No, 1 = Yes) and depression (0 = No, 1 = Yes).We also derived some lifestyle behaviour variables from the following questions: ‘how many servings of fruits, and vegetables do you eat on a typical day? And ‘Have you ever smoked tobacco or used smokeless tobacco? (recoded 0 = No, 1 = Yes), and Have you ever consumed a drink that contains alcohol? (recoded 0 = No, 1 = Yes). Health-seeking behaviour characterised by the number of clinical visits (recoded 0 = not at all, 1 = once/twice, 2 = three to six times, 3 = more than six times) was also included as an independent variable.

### Data analysis

We used STATA Version 14 as the tool for data analyses. Descriptive statistics were used to summarise hypertension status and its correlates. Chi-square test were used to test for differences between categorical variables. Binary logistic regression analysis was used to examine variables associated with hypertension. In all, four Models were fitted in the study. Model I introduced only socio-demographic factors (age, sex, education, employment, wealth status and body mass index). Model 2 adjusted for comorbidities (depression, heart disease, arthritis, asthma, diabetes, cancer and stroke). Model 3 varies from Model 1 & 2 based on the inclusion of lifestyle behaviour (tobacco and alcohol consumption, and fruit and vegetable consumption), and the complete model includes health-seeking (times visited the clinic in the last 12 months) in addition to all variables in preceding models (I-IV).

### Ethical approval

This study followed the Declaration of Helsinki. The Ethics Review Committee of the World Health Organization, Geneva, Switzerland, approved the South Africa-SAGE Well-Being of Older People Study (WOPS) Wave 2. All participants signed a written informed consent form. The authors of this paper were not directly involved in the data collection operations. All methods were performed in accordance with the relevant guidelines and regulations. We requested access to the data at: http://www.who.int/healthinfo/sage/cohorts/en/.

## Results

### Background characteristics by hypertension status

Table 1 presents proportions of respondents’ hypertension status by, socio-demographic, comorbidities, lifestyle behaviour and health-seeking variables. Most of the respondents were aged 50–59 years and predominantly females. Predominantly, the participants were widowed, had basic education, unemployed, and with a normal BMI. Overall, out of the 518 respondents, 50.1% of them were hypertensive. The prevalence of hypertension was higher among females (58.0%), those aged 80 years and above (65.0%), ever been diagnosed with stroke (71.4%), and ever diagnosed with diabetes (74.4%). The prevalence of hypertension was higher among those who visited the clinic 3–6 times within the last 12 months prior to the survey (56.8%).

**Table 1:** Background characteristics by hypertension status

Covariates	Frequency	Hypertensive Status				X^2^	*p*-value
		**Non-hypertensive**		**Hypertensive**			
		**%**	**(n)**	**%**	**(n)**		
Overall	517	49.9	258	50.1	259		
***Socio-demographics***							
**Age**						10.44	0.02
50–59	249	56.6	141	43.4	108		
60–69	149	47.7	71	52.4	78		
70–79	79	41.8	33	58.2	46		
80 +	40	35.0	14	65.0	26		
**Sex**						44.95	0.00
Male	118	77.1	91	22.9	27		
Female	400	42.0	168	58.0	232		
**Marital status**						2.38	0.50
Married	136	54.4	74	45.6	62		
Separated/divorced	37	54.0	20	46.0	17		
Never married	135	50.4	68	49.6	67		
Widowed	209	46.4	97	53.6	112		
**Education**						5.72	0.06
No education	250	46.8	117	53.2	133		
Basic	256	51.6	132	48.4	124		
Secondary and above	11	81.8	9	18.2	2		
**Employment**						7.44	0.01
Not working	468	48.1	225	51.9	243		
Working	48	68.8	33	31.2	15		
**Body mass index**						8.46	0.04
Underweight	30	63.3	19	36.7	11		
Normal	159	58.5	93	41.5	66		
Overweight	119	51.3	61	48.7	58		
Obese	172	44.2	76	55.8	96		
**Wealth status**						0.69	0.95
Poorest	102	49.0	50	51.0	52		
Poorer	102	51.0	52	49.0	50		
Middle	101	49.5	50	50.5	51		
Richer	109	52.3	57	47.7	52		
Richest	94	46.8	44	53.2	50		
**Comorbidity**							
*Ever diagnosed with depression*						1.17	0.28
No	483	50.5	244	49.5	239		
Yes	32	40.6	13	59.4	19		
*Ever diagnosed with heart disease*						0.42	0.52
No	507	50.3	255	49.7	252		
Yes	10	40.0	4	60.0	6		
*Ever diagnosed with arthritis*						2.83	0.09
No	395	52.1	206	47.9	189		
Yes	122	43.4	53	56.6	69		
*Ever diagnosed with asthma*						2.06	0.15
No	491	49.3	242	50.7	249		
Yes	25	64.0	16	36.0	9		
*Ever diagnosed with diabetes*						11.28	0.00
No	474	52.3	248	47.7	226		
Yes	43	25.6	11	74.4	32		
*Ever diagnosed with cancer*						2.73	0.10
No	510	50.6	258	49.4	252		
Yes	6	16.7	1	83.3	5		
*Ever diagnosed with stroke*						4.09	0.04
No	495	51.1	253	48.9	242		
Yes	21	28.6	6	71.4	15		
**Lifestyle behaviour**							
Fruit consumption						0.38	0.83
< 2	145	48.3	70	51.7	75		
2	194	49.0	95	51.0	99		
3 +	155	51.6	80	48.4	75		
Vegetable consumption						2.20	0.33
< 2	283	48.1	136	51.9	147		
2	138	49.3	68	50.7	70		
3 +	97	56.7	55	43.3	42		
Tobacco consumption						6.77	0.01
No	450	48.0	216	52.0	234		
Yes	66	65.2	43	34.9	23		
Alcohol consumption						8.80	0.00
No	405	46.7	189	53.3	216		
Yes	112	62.5	70	37.5	42		
***Health seeking***							
Times visited the clinic in last 12 months						8.69	0.03
No visit	22	72.7	16	27.3	6		
Once/twice	72	56.9	41	43.1	31		
3 – 6 times	148	43.2	64	56.8	84		
More than six times	239	49.0	117	51.0	122		

### Binary logistic regression results of associated factors of hypertension

Table 2 shows the results from the binary logistic regression showing the factors associated with hypertension among PLHIV. In Model IV, which is the final model, age, sex, ever diagnosed with stroke and number of times visited clinic were the factors that were associated with hypertension among PLHIV. Compared to PLHIV aged 50–59, those aged 60–69 [AOR = 2.2; CI = 1.30,3.84], 70–79 years [AOR = 2.8; CI = 1.37,5.82], and 80 + [AOR = 4.9; CI = 1.68,14.05] had higher risk of hypertension. Concerning sex, females living with HIV were more likely [AOR = 5.5; CI = 2.67,11.12] than males to have hypertension. Persons ever diagnosed with stroke were more likely [AOR = 3.3; CI = 1.04,10.65] to have hypertension as compared to their counterparts who have never been diagnosed with stroke. Compared to PLHIV who had no clinic visits, those who visited the clinic 3–6 times [AOR = 5.3; CI = 1.35,21.01] or more than six times [AOR = 5.5; CI = 1.41,21.41] were more likely to have hypertension.

**Table 2:** Binary logistic regression results of associated factors of hypertension

Explanatory variables	Model I	Model II	Model III	Model IV
	OR (95% CI)	AOR (95% CI)	AOR (95% CI)	AOR (95% CI)
***Socio-demographics***				
**Age**				
50–59	Ref	Ref	Ref	Ref
60–69	1.6 [0.99,2.48]	1.7* [1.03,2.69]	1.8* [1.08,2.89]	2.2** [1.30,3.84]
70–79	2.1* [1.11,4.04]	2.3* [1.18,4.49]	2.3* [1.17,4.68]	2.8** [1.37,5.82]
80 +	2.6* [1.11,5.88]	2.8* [1.19,6.58]	3.0* [1.20,7.55]	4.9** [1.68,14.05]
**Sex**				
Male	Ref	Ref	Ref	Ref
Female	5.9*** [3.20,10.74]	5.9*** [3.07,11.22]	5.5*** [2.75,10.85]	5.5*** [2.67,11.12]
**Marital status**				
Married	Ref	Ref	Ref	Ref
Separated/ divorced	0.6 [0.24,1.50]	0.6 [0.22,1.41]	0.6 [0.23,1.66]	0.7 [0.27,2.00]
Never married	0.8 [0.47,1.52]	0.9 [0.47,1.62]	0.9 [0.47,1.73]	0.8 [0.40,1.58]
Widowed	0.8 [0.48,1.36]	0.8 [0.48,1.43]	0.9 [0.51,1.60]	0.8 [0.45,1.55]
**Educational level**				
No education	Ref	Ref	Ref	Ref
Basic	0.8 [0.56,1.27]	0.8 [0.54,1.26]	0.8 [0.54,1.28]	0.8 [0.51,1.27]
Secondary and above	0.3 [0.04,1.70]	0.2 [0.02,2.06]	0.2 [0.02,2.41]	0.2 [0.02,1.82]
**Employment**				
Not working	Ref	Ref	Ref	Ref
Working	0.7 [0.34,1.36]	0.6 [0.31,1.34]	0.6 [0.28,1.28]	0.9 [0.39,1.99]
**Body mass index**				
Underweight	Ref	Ref	Ref	Ref
Normal	0.9 [0.37,2.42]	0.9 [0.35,2.75]	1.0 [0.35,3.09]	0.9 [0.28,2.92]
Overweight	1.0 [0.40,2.75]	1.0 [0.36,1.80]	1.1 [0.36,3.47]	1.1 [0.32,3.59]
Obese	1.2 [0.47,3.09]	1.2 [0.44,3.51]	1.2 [0.42,3.66]	1.2 [0.37,3.89]
**Wealth status**				
Poorest	Ref	Ref	Ref	Ref
Poorer	0.9 [0.50,1.74]	0.8 [0.46,1.61]	0.7 [0.37,1.43]	0.6 [0.29,1.25]
Middle	1.0 [0.55,1.99]	0.9 [0.48,1.81]	0.9 [0.42,1.71]	0.6 [0.30,1.38]
Richer	0.7 [0.37,1.29]	0.7 [0.37,1.29]	0.6 [0.31,1.20]	0.5 [0.26,1.10]
Richest	1.1 [0.53,2.09]	1.0 [0.49,2.05]	1.0 [0.47,2.12]	0.7 [0.32,1.74]
***Comorbidity***				
Ever diagnosed with depression				
No		Ref	Ref	ref
Yes		1.5 [0.68,3.43]	1.5 [0.65,3.60]	1.4 [0.57,3.29]
Ever diagnosed with heart disease				
No		Ref	Ref	Ref
Yes		1.0 [0.21,5.05]	1.3 [0.23,7.17]	1.7 [0.22,13.44]
Ever diagnosed with arthritis				
No		Ref	Ref	Ref
Yes		1.3 [0.79,2.08]	1.4 [0.84,2.26]	1.5 [0.87,2.62]
Ever diagnosed with asthma				
No		Ref	Ref	Ref
Yes		0.7 [0.21,2.07]	0.5 [0.16,1.60]	0.5 [0.16,1.56]
Ever diagnosed with diabetes				
No		Ref	Ref	Ref
Yes		2.1 [0.93,4.60]	2.0 [0.88,4.69]	2.2 [0.94,5.28]
Ever diagnosed with cancer				
No		Ref	Ref	Ref
Yes		5.5 [0.90,33.20]	6.2 [0.98,39.35]	5.7 [0.75,42.79]
Ever diagnosed with stroke				
No		Ref	Ref	Ref
Yes		1.9 [0.65,5.65]	2.6 [0.84,8.22]	3.3* [1.04,10.65]
***Lifestyle behaviour***				
Fruit consumption				
< 2			Ref	Ref
2			1.12 [0.65,1.94]	1.0 [0.54,1.74]
3 +			0.9 [0.49,1.50]	0.9 [0.51,1.72]
Vegetable consumption				
< 2			Ref	Ref
2			0.9 [0.53,1.46]	0.7 [0.43,1.25]
3 +			0.6 [0.33,1.12]	0.6 [0.33,1.20]
Tobacco consumption				
No			Ref	Ref
Yes			1.1 [0.49,2.31]	1.0 [0.44,2.29]
Alcohol consumption				
No			Ref	Ref
Yes			0.9 [0.51,1.76]	1.8 [0.59,2.37]
***Health seeking***				
Times visited the clinic in last 12 months				
No visit				Ref
Once/twice				1.9 [0.46,7.76]
3 – 6 times				5.3* [1.35,21.01]
More than six times				5.5* [1.41,21.41]
**Constant**	0.23	0.19	0.22	0.06
**Model fitness**				
Prob > chi2	< 0.001	< 0.00	< 0.00	< 0.00
AIC	612.29	606.23	594.15	546.44
Pseudo R^2^	0.11	0.12	0.13	0.15
N	518	518	518	518

^*^p < 0.05, **p < 0.01, ***p < 0.001; *ref* reference category, *OR* odds ratio, *CI* confidence interval

## Discussion

The study reveals that there is a high prevalence of hypertension (50.1%) among PLHIV in South Africa. The estimated prevalence is higher than the 14.3% prevalence that was reported by Chiwandire et al. [8]. This prevalence is further higher than the estimated prevalence in other African countries such as Ghana (30.8%) [24], and Ethiopia (12.7%) [17]. It is worth noting that unlike previous studies, this study population is limited to elderly PLHIV. The sharp difference between the prevalence found in this study when compared to other studies, clearly indicates that the prevalence of hypertension in PLHIV increases with increasing age. Our study underscores the urgency and need for the South African government to prioritise and strengthen the healthcare system to integrate hypertension management into HIV care. Hypertension advocacy would have to be part of the basic service package provided to PLHIV in South Africa. This may be beneficial in the long run to reduce the prevalence of hypertension among this cohort.

Concerning the factors associated with hypertension among PLHIV, we found sex differences in the risk of hypertension. Older females living with HIV were five times more likely than their male counterparts to have hypertension. Similar findings have been reported in studies conducted among the general South African HIV population [8]. The findings are further substantiated by earlier studies that found similar sex variations in the risk of hypertension among PLHIV [25, 26]. A plausible explanation for the sex differences is that, unlike men, women go through a series of body changes such as menopause. After menopause, as is in the case of older women, there is endogenous oestrogen withdrawal which exacerbates the likelihood of post-menopausal hypertension [27]. During pregnancy, women sometimes face gestational hypertension and eclampsia [28].

Ageing was another factor that increased the risk of hypertension among South African older PLHIV. Persons aged 80 years and older had the greatest odds of having hypertension compared to those aged 50–59 years. This finding mirrors that of previous studies conducted in Ghana [24], South Africa [8], Nigeria [15], Malawi [16] and Ethiopia [17]. As opined by Fahme, Bloomfield and Peck [29], ageing is characterised by gradual vascular stiffening which significantly increases blood pressure, hence, exacerbating the risk of hypertension. Such biological effects of increased arterial resistance and vascular stiffening, may thus, explain why ageing significantly increases the risk of hypertension in PLHIV. The findings imply that age can be a marker for beginning hypertension management during HIV care. Standard modules for mandatory hypertension management sessions for older PLHIV would be necessary for reducing the risk of hypertension among older PLHIV.

Our study reveals that older PLHIV who had ever been diagnosed with stroke were three times more likely to have hypertension as compared to their counterparts who have never been diagnosed with stroke. The present study is consistent with findings from a hospital-based survey that showed that hypertension increased among persons who have ever been diagnosed with stroke for the first time [30]. It is unclear how and why the risk of hypertension is high among persons who have ever been diagnosed with stroke. A related study [31] has shown that substantial proportion of hypertensive go unaware until a stroke occurs for the first time. Although not significant in the final regression model, 74.4% of persons ever diagnosed of diabetes were hypertensive. Ferrannini and Cushman [32] have postulated that the high prevalence of hypertension among person diagnosed with diabetes may be due to biological pathways such as, *“insulin resistance in the nitric-oxide pathway; the stimulatory effect of hyperinsulinaemia on sympathetic drive, smooth muscle growth, and sodium–fluid retention; and the excitatory effect of hyperglycaemia on the renin–angiotensin–aldosterone system”*. Therefore, older PLHIV who get diagnosed with diabetes would have initiate preventive and control interventions for hypertension. Relatedly, we observed that older PLHIV who often visited the clinic were more likely to have hypertension. Thus, through frequent clinic visits, PLHIV have the opportunity to undergo hypertension screenings and gain information about hypertension. Consequently, they become more likely to get to know about their hypertensive status.

Our findings call for the integration of hypertension management into ongoing HIV care services across all levels of the healthcare architecture. These could be enhanced by having hospital guidelines that integrate HIV and hypertension care along healthcare continuum. National advocacy and campaign could be championed to accelerate efforts to have hypertension management integrated in all aspect of healthcare to older PLHIV. There is also the need to sustain health education and promotion programmes that are tailored to the needs of women if efforts are to be made to reduce the prevalence of hypertension and HIV amongst them.

### Strengths and limitations

Our study draws its conclusions from a representative sample size of older PLHIV. Hence, we are able to generalise our findings to all older PLHIV in South Africa. Also, the questionnaires and methods of data collection used by the WHO WOPS has been validated, thereby ensuring the reliability of our findings. Nevertheless, there are some limitations that must be taken into account when interpreting the findings. We relied on a secondary data that used cross-sectional design. As such, we are unable to establish causal inferences in the risk factors of hypertension among older PLHIV. Also, the source data does not capture evidence on which health outcome occurred prior to the other. For instance, hypertension is a major risk factor for stroke. Our findings that the risk of hypertension is high among persons who have ever been diagnosed with stroke could therefore be due to the fact that hypertension occurred prior to stroke events.

## Conclusion

In this study, we examined the prevalence and factors associated with hypertension among older people living with HIV in South Africa. We conclude that more than half of South African older PLHIV are hypertensive. Also, the factors associated with hypertension among older PLHIV are age, sex, ever diagnosed with stroke and number of times visited the clinic. Integration of HIV care and hypertension management, and advocacy in HIV care is urgently needed in South Africa in order to accelerate reductions in the prevalence of hypertension among older PLHIV, as well as enhance South Africa’s capacity to attain the SDG targets, particularly SDG 3.3.

## Data Availability

The data used to support the findings of this study is available from the corresponding author upon request. Data is available at the WHO SAGE Wave 2 office and through the WHO website http://www.who.int/healthinfo/sage/cohorts/en/.

## Abbreviations

ACDIS: Africa Centre Demographic Information System
ART: Anti-retroviral therapy
HIV: Human immunodeficiency virus
PLHIV: People living with HIV
SDGs: Sustainable Development Goals
SSA: Sub-Saharan Africa
WOPS: Well-Being of Older People Study
